# MS-Based Metabolite Profiling of Aboveground and Root Components of *Zingiber mioga* and *Officinale*

**DOI:** 10.3390/molecules200916170

**Published:** 2015-09-03

**Authors:** Ji Soo Han, Sunmin Lee, Hyang Yeon Kim, Choong Hwan Lee

**Affiliations:** Department of Bioscience and Biotechnology, Kon-Kuk University, Seoul 143-701, Korea; E-Mails: loveheartlovone@gmail.com (J.S.H.); duly123@naver.com (S.L.); festival kim@naver.com (H.Y.K.)

**Keywords:** *Zingiber mioga*, *Zingiber officinale*, antioxidant activity, Zingiberaceae, metabolomics, UPLC-Q-TOF-MS, GC-TOF-MS

## Abstract

*Zingiber* species are members of the Zingiberaceae family, and are widely used for medicinal and food purposes. In this study aboveground and root parts of *Zingiber mioga* and *Zingiber* *officinale* were subjected to metabolite profiling by ultra-performance liquid chromatography-quadrupole-time-of-flight mass spectrometry (UPLC-Q-TOF-MS) and gas chromatography time-of-flight mass spectrometry (GC-TOF-MS) in order to characterize them by species and parts and also to measure bioactivities. Both primary and secondary metabolites showed clear discrimination in the PCA score plot and PLS-DA by species and parts. Tetrahydrocurcumin, diarylheptanoid, 8-gingerol, and 8-paradol were discriminating metabolites between *Z. mioga* and *Z*. *officinale* that were present in different quantities. Eleven flavonoids, six amino acids, six organic acids, four fatty acids, and gingerenone A were higher in the aboveground parts than the root parts. Antioxidant activities were measured and were highest in the root part of *Z. officinale*. The relatively high contents of tetrahydrocurcumin, diarylheptanoid, and galanganol C in the root part of *Z. officinale* showed highly positive correlation with bioactivities based on correlation assay. On the basis of these results, we can suggest different usages of structurally different parts of *Zingiber* species as food plants.

## 1. Introduction

Species of the genus of *Zingiber* are members of the Zingiberaceae (ginger) family. They are widely known for their important use as ornamental, spice, and medicinal plants. In Asian countries, they are extensively used as traditional medicines to treat headaches, nausea and colds. In the herbal medicinal practice of Western countries, they are used for the treatment of muscular discomfort, arthritis, rheumatic disorders, catarrh, toothache, asthma, stroke, gingivitis, constipation, nervous diseases and diabetes [1,2,3]. The different parts of ginger species have different properties, which have determined the use of a particular ginger species. Most ginger family plants are aromatic perennial herbs with the characteristic of growing horizontal or fibrous rhizomes. Among the many other species of *Zingiber* plants two species are majorly found in Korea; Z*ingiber mioga Rosc.* Known as myoga ginger [4] and Z*ingiber Officinale Rosc.* ginger [1]. *Zingiber* *officinale* is the best known *Zingiber* plant in the ginger family and is also referred to as garden ginger or ginger. Extracted essential oils and oleoresins from the *Z. officinale* rhizome are invaluable chemical substances illustrating the pungency and feature of ginger flavor [5]. The seeds of *Z. officinale* contains volatile oils (shogaols, paradols, gingerols, and gingerdiols) which are effective against pain and swelling [6]. The extracts obtained from root of *Z. officinale* contain polyphenol compounds (gingerol, shogaol, paradol, and those derivatives) which have antioxidant activity [3]. Z*. officinale* has been cultivated for years and widely used for medicinal purposes and as a spice additive to food [2]. The flower bud of *Z. mioga* which grows at ground level and has a unique odor, is used widely as a spice and herbal medicine in Japan, China and Korea [7]. For medicinal use the subterranean stems and seeds of *Z. mioga* are often used in Chinese herbal medicines [1]. In particular the flower buds of *Z. mioga* are used as a spice in foods and as an ingredient in pickles because it has a unique odor and similar taste to *Z. officinale* ginger. The subterranean stem and young flower buds of *Z. mioga* contain zingerene, zingerone, shogaol, and β-phellandrene which can used to cure menstrual irregularity, leucorrhea, odynolysis, heart disease, and eye inflammation. They can also be used as an expectorant [8]. 

There has been previous research into the non-polar metabolites, such as essential oils and oleoresins [9] and quantitative analysis of the pungent principles of the Zingiberaceae family (curcuminoids, gingerol, shogaol, and paradol) were undertaken [10,11,12,13]. Also targeted analyses of metabolites (curcuminoids, terpenoids, gingerol and its derivatives) or their biological effects [10] were performed. Many other studies have focused on measuring the antioxidant activity, total flavonoid and phenol contents of different parts in Zingiberaceae plants but did not present evidence for any correlation between their differential secondary metabolites and their bioactivities. 

From the perspective of providing a global view of metabolism and characterization by its silent phenotype, we used metabolomics which is an independent part of systems biology [14]. Today, it has been applied to many field of studies in microorganisms [15], plants [16], environmental [17], and mammalian systems [18]. The powerful UPLC-Q-TOF-MS and GC-TOF-MS analytical platforms are often used in plant metabolomics to profile differential metabolites. We have now used them to profile differential metabolites in different structural parts of *Z. officinale* and *Z. mioga* and to correlate them with their bioactivities. 

The aim of this study was to differentiate two different *Zingiber* species using untargeted metabolomics. The two species have medicinal dietary applications of different structural parts. Their uses are dependent on the properties of the different structural parts of each plant. To the best of our knowledge no other studies have used non-targeted metabolomics to compare different parts of *Zingiber* species. Different from previous studies, we investigated two *Zingiber* species’ bioactivities (antioxidants, total phenol content (TPC), and total flavonoid content (TFC)) and analyzed differential metabolites using UPLC-Q-TOF-MS and GC-TOF-MS combined with multivariate analysis. We suggest potential biomarkers correlated with bioactivities that can discriminate *Zingiber* species and different parts. Information from our research will improve the selection of appropriate different parts or species for dietary or medicinal applications that are dependent on bioactivities.

## 2. Results and Discussion

### 2.1. Primary Metabolites Discriminating Z. mioga and Z. officinale Analyzed by GC-TOF-MS

**Figure 1:**
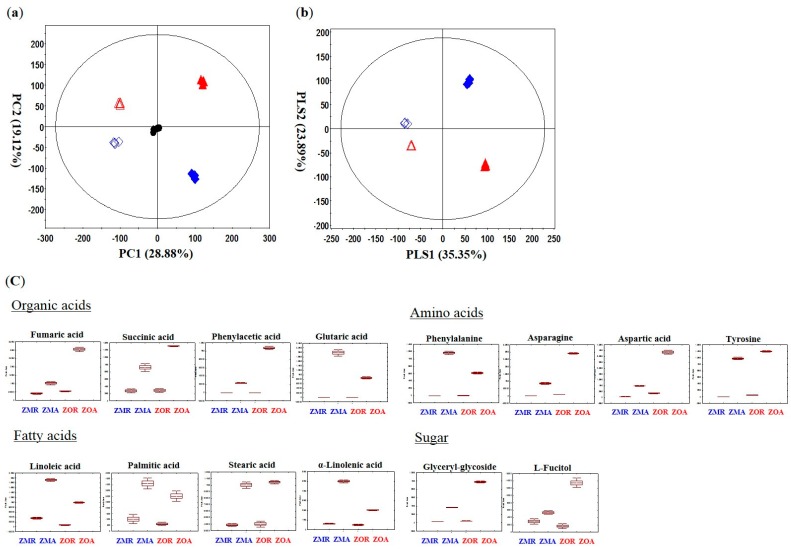
Principal component analysis score plot (PCA) (**a**), Partial least-squares discriminant analysis (PLS-DA) (**b**), and box whisker plots (**c**) of *Z. officinale* and *Z*. *mioga* with different structural parts analyzed by GC-TOF-MS. ▲: ZMR (*Zingiber mioga* Thunb. Root part), △: ZMA (*Zingiber mioga* Thunb. Aboveground part), ♦: ZOR (*Zinger Officinale* Thunb*.* Root part), ◊: ZOA (*Zingiber Officinale* Thunb. Aboveground part), and ●: Quality Control.

Multivariate analysis was performed with the data obtained from the GC-TOF-MS analysis to specify different metabolites and suggest comparisons between *Z. mioga* and *Z. officinale* and by different parts (*Zingiber mioga* Root (ZMR), *Zingiber mioga* Aboveground (ZMA), *Zingiber Officinale* Root (ZOR), and *Zingiber Officinale* Aboveground (ZOA)). Correcting the error in the instrumental analysis, quality control samples were obtained by pooling. The quality control samples clustered well in the PCA score plot, meaning the data was reliable. In the PCA score plot, the two *Zingiber* species showed significant separations between *Z. mioga* and *Z. officinale* and their different parts. The aboveground and root part were discriminated by PC1 (28.88%) and *Z. mioga* and *Z. officinale* were discriminated by PC2 (19.22%) (Figure 1a,b). From the PLS-DA of the four groups of aboveground and root parts of *Zingiber* species, a total of 34 significant metabolites were selected on the basis of VIP values (VIP > 0.7) and *p*-values (*p*-value < 0.05) [19]. Seventeen organic acids, seven amino acids, five fatty acids, and five sugars were identified and are listed in Table S1 (Supplementary Information). Metabolites discriminating between aboveground and root parts by VIP 1 were hexanoic acid, acetic acid, pyruvic acid, butanoic acid, d-2-aminobutyric acid, benzoic acid, phenylacetic acid, succinic acid, fumaric acid, penitol, glycine, l-aspartic acid, l-threonic acid, l-tyrosine, linoleic acid, stearic acid, arachidic acid, palmitic acid, d-tagatose, d-galactose, l-fucitol, and glycerylglycoside. Metabolites discriminating between *Z. mioga* and *Z. officinale* by VIP 2 were propanoic acid, ethylmalonic acid, and serine. Fourteen metabolites with clear patterns were expressed by box whisker plots in order to compare their relative contents by parts and species (Figure 1c). 

Organic acids such as butanoic acid, D-2-aminobutryric acid, phosphoric acid, benzoic acid, phenylacetic acid, succinic acid, fumaric acid, glutaric acid, *meso*-erythritol, and glycerol showed higher contents in the aboveground part of *Zingiber* species than the root part [20]. Citric acid, succinic acid, and fumaric acid, which are known to be transported from the root by the plant’s innate transport system to the aboveground part, were abundant in the aboveground part of *Zingiber* species where photosynthesis is active [21,22,23]. Amino acids, such as l-aspartic acid, l-threonic acid, phenylalanine, l-asparagine and l-tyrosine seemed to have higher contents in aboveground parts than root parts of *Zingiber* species. Phenylalanine is the precursor of pungent component such as the curcuminoids in the Zingiberaceae family [24]. Aspartic acid levels were high in aboveground part as the precursor of asparagine provides a nitrogen source in response to sugar starvation. Metabolism transforms aspartic acid into asparagine which showed high contents in the aboveground parts of *Zingiber* species [25], where it accumulates in response to drought and rewatering of the plant, which may be a stress and threat to the plant [26]. The aboveground part can be more effective when processed as food for good quality [27] because the amino acids contents are generally higher in the aboveground parts of both *Zingiber* species. 

Fatty acids, for example linoleic acid, α-linolenic acid, stearic acid, and palmitic acid, seemed to have higher contents in the aboveground parts than root parts. Essential fatty acids which cannot be synthesized endogenously by humans or mammals such as α-linolenic acid and linoleic acid must be obtained from other sources like foods derived from plants [28]. Environmental (pathogen attack, touch response, drought and UV light) and developmental (pollen development, tuberization, and storage) stress or wounding could induce the primary and secondary metabolism of plant and lead to the accumulation of fatty acids like linoleic and linolenic acid in the aboveground parts of *Z. officinale* and *Z*. *mioga* as part of the defense system [29]. Our results were consistent with previous research on amino acids and fatty acids, which showed that they were more abundant in the aboveground parts than the root parts [30,31]. 

Sugars, such as d-tagaose, and glycerylglycoside, were selected as discriminating metabolites in the different parts and showed higher contents in the aboveground parts of *Zingiber* species, particularly in *Z. officinale*. Photosynthesis is active in the aboveground part where sugar-producing organs are found. Sugars accumulate as the product of photosynthesis and in the night respiration process in the aboveground parts rather than the root parts of *Zingiber* species [32]. 

Considering the relative contents of metabolites by species and in their different parts, amino acids, organic acids and fatty acids levels were higher in aboveground parts than the root parts of *Zingiber*, indicating the nutritional value and distribution of phytochemicals by different parts in the same species. This information will enable manufacturers to identify the different parts of *Zingiber* species that are best suited for usage as functional foods or medicines. 

### 2.2. Secondary Metabolites Discriminating Z. mioga and Z. officinale Analyzed by UPLC-Q-TOF-MS

**Figure 2:**
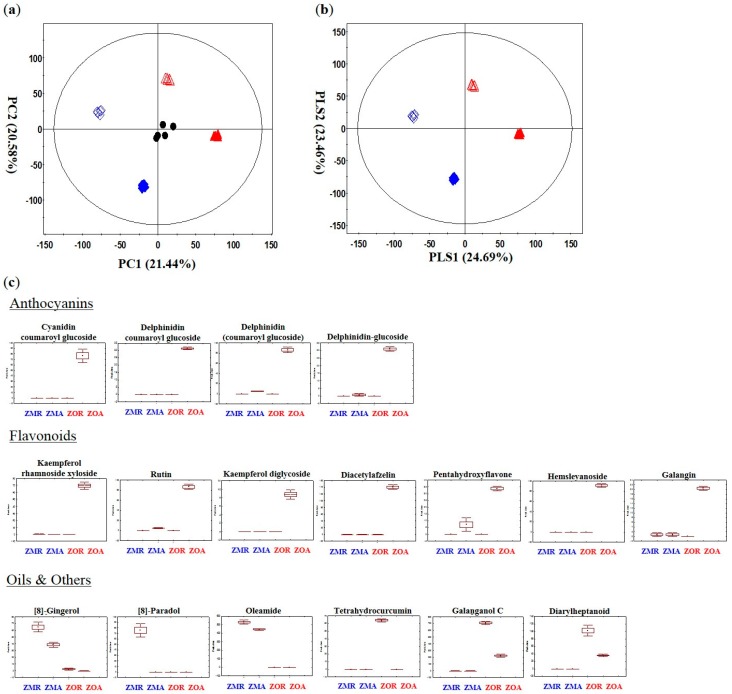
Principal component analysis score plot (PCA) (**a**), Partial least-squares discriminant analysis (PLS-DA) (**b**), and box whisker plot (**c**) of *Z. officinale* and *Z. mioga* with different parts analyzed by UPLC-Q-TOF-MS. ▲: ZMR (*Zingiber mioga* Thunb. Root part), △: ZMA (*Zingiber mioga* Thunb. Aboveground part), ♦: ZOR (Z*ingiber Officinale* Thunb*.* Root part), ◊: ZOA (*Zingiber Officinale* Thunb. Aboveground part), and ●: Quality Control.

Multivariate analysis was applied to the UPLC-Q-TOF-MS data set to investigate the secondary metabolites that distinguish between the two *Zingiber* species, and with their different structural parts. Clear clustering of *Z. officinale* and *Z*. *mioga*, aboveground and root parts were visualized in the PCA and PLS-DA score plots (Figure 2a,b). The clustering pattern of the quality control sample showed that there was a low possibility of instrument analysis and data processing errors. The PCA score plot of the UPLC-Q-TOF-MS data set clustered each species and the different structural parts and the resulting patterns were similar to the PCA score plot of the GC-TOF-MS data. *Z. officinale* and *Z*. *mioga* were clearly separated by PC 1 (21.44%) and the root and aboveground parts were clearly separated by PC 2 (20.58%). A total of 55 secondary on the basis of VIP values (VIP > 0.7) and *p*-values (*p*-value < 0.05). Identification of the differentially expressed metabolites was carried out using high-resolution mass data (i-fit, ppm), and by searching and matching with reference databases [12,13,33,34,35,36,37] (Table S2, Supplementary Information). Metabolites discriminating *Z. mioga* and *Z. officinale* by VIP 1 were tetrahydrocurcumin, aminooctadecenetriol, 8-gingerol, labdene-diol, [8]-paradol, and palmitic acid. By VIP 2, metabolites discriminating root and aboveground part were isoleucine, phenylalanine, 2-(methylamino)benzoic acid, tryptophan, kaempferol diglycoside, rutin, kaempferol rhamnoside xyloside, diacetylafzelin, pentahydroxyflavone, hemslyanoside, galangin, cyanidin coumaroyl glucoside, delphinidin coumaroyl glucoside, delphinidin, 6-gingerdiol, trihydroxy octadecenoic acid, galaganol C, gingerenone A, curcumadiol, oxo-octadecenoic acid, amino-eicosanediol, aminomethylnondecanetriol, amino-heneicosene diol, halaminol A, and palmitoleamide. Seventeen metabolites showing discriminative pattern were drawn as box whisker plots to compare their relative contents by different parts and species (Figure 2c). Seven flavonoids (kaempferol diglycoside, rutin, kaempferol rhamnoside xyloside, diacetylafzelin, pentahydroxyflavone, hemslyanoside, and galangin) and four anthocyanins (cyanidin coumaroyl glucoside, delphinidin coumaroyl glucoside, delphinidine glucoside, and delphinidin) showed a pattern of higher contents in aboveground parts than the roots of *Zingiber* species*.* Furthermore, they were also higher in the aboveground parts of *Z. officinale* than in *Z. mioga* [33,34]. The aboveground parts of *Zingiber* species are where the density and intensity of light are focused which can explain the higher contents of flavonoids and anthocyanins in the aboveground parts than the root parts [36]. Flavonoids and anthocyanins are the final products produced in the flavonoid biosynthesis pathway and they protect the plant from biotic and abiotic stresses [35]. Gingerglycolipid A, 8-gingerol, labdene diol, 8-paradol, palmitic acid, and oleamide were discriminating metabolites between *Z. officinale and Z. mioga* and showed the highest contents either in the aboveground parts or root parts of *Z. mioga*. Discriminating *Zingiber* species and at the same time showing the highest contents in the root part of *Z. officinale* were tetrahydrocurcumin and diarylhetapnoid. Pungent components of the Zingiberaceae family such as galanganol C, gingerenone A, and curcumadiol discriminated between different parts of *Zingiber* but didn’t show a consistent relative pattern. Gingerglycolipid A which belongs in the monoacyldigalactosylglycerol class has anti-ulcer effects in the stomach of animals [38]. Tetrahydro-curcumin which is a pungent component in *Curcuma* species, is known to have anti-cancer and anti-angiogenic effects [39]. Both of these metabolites were mostly detected in the roots and aboveground parts of *Z. officinale* (Figure 2c) [38,39]. Likewise two other major components of *Zingiber* species: 8-gingerol and 8-paradol, which have efficacy in anti-inflammatory, antibacterial, and antitumorigenic susbtances, are the chain branched forms of gingerol and paradol, respectively, and were mostly detected in *Z. mioga*, specifically in the root part (Figure 2b) [38,39,40]. Polyphenols which have antioxidant activity and anti-inflammatory effects like tetrahydrocurcumin, diarylheptanoid, 8-paradol and 8-gingerol were major metabolites discriminating *Z. mioga* and *Z. officinale.* Tetrahydrocurcumin as a diarylheptanoid and 8-gingerol and 8-paradol, which are gingerols, are part of the phenylpropanoid pathway [41]. These results for the metabolites mentioned above may change the way various *Zingiber* species and their different parts can be used as nutritional plants and functional foods. 

### 2.3. Correlation between the Significant Metabolites and Bioactivities

**Figure 3:**
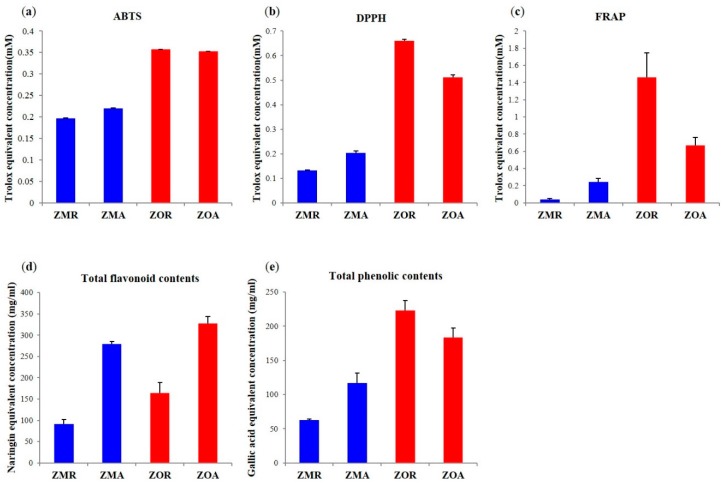
Antioxidant activity assay, ABTS (**a**), DPPH (**b**), and FRAP(**c**), total flavonoid contents (**d**) and total phenolic contents (**e**) of *Z. officinale* and *Z. mioga* with the two different structural parts.

To compare the bioactivities with *Z. mioga* and *Z*. *officinale* dependent on their different parts, antioxidant activity assays were undertaken using the ABTS, DPPH, and FRAP methods, while TFC, and TPC assays were also performed (Figure 3). The highest antioxidant activity corresponded to the root parts of *Z. officinale*, followed by the aboveground parts of *Z. officinale.* The antioxidant activity results appeared to be higher in *Z. officinale* than *Z. mioga*. Particularly, root parts of *Z. officinale*, showed the highest antioxidant activity. The results of the antioxidant activity assays (ABTS, DPPH, and FRAP) and TPC assay were consistent in the following order: ZOR > ZOA > ZMA > ZMR, but the TFC assay results showed the both the aboveground parts of *Zingiber* species were higher than the root part in the following order: ZOA > ZMA > ZOR > ZMR (Figure 3). Other studies have already shown the relation with TPC and TFC with antioxidant activity (ABTS, DPPH, and FRAP). Flavonoids and polyphenols are known to have considerable bioactivity, e.g., as antioxidants [42,43]. High antioxidant activity was shown in aboveground and root part of *Z. officinale* which is probably due to the presence of metabolites having potential antioxidant activity [2]. 

**Figure 4:**
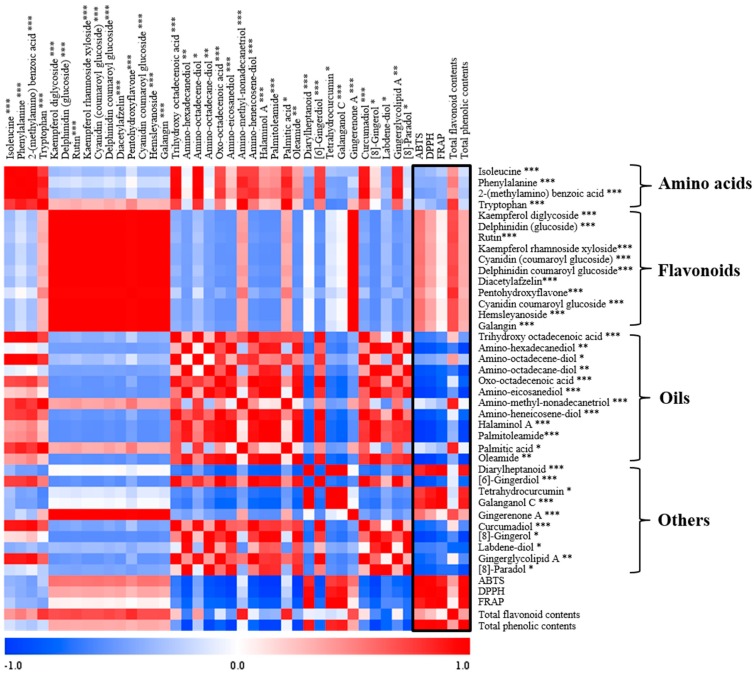
Correlation map of the metabolites analyzed by UPLC-Q-TOF-MS with antioxidant activity, TPC, and TFC. Each square indicates r (Pearson’s correlation coefficient values for pairs of metabolite or antioxidant activity values). The red color represents a positive (0 < r < 1) correlation and the blue color represents a negative (−1 < r < 0) correlation. In the tentative compounds, * represents the metabolites differentiated by VIP value 1, ** represents metabolites differentiated both by VIP value 1 and 2, and *** represents metabolites differentiated by VIP value 2.

*Z. officinale* has already been reported to have antioxidant activity and previous research was performed in many other directions such as targeted analysis of pungent principles of *Z. officinale* such as gingerol, shogaol, paradol, and zingerone were performed and their potential antioxidant activity were investigated [3,6]. Different metabolites contents in different parts of *Zingiber* species caused the observed differences in the bioactivities. In order to observe the relationship between bioactivities and discriminating metabolites, a correlation assay was performed. As Figure 4 shows, the flavonoids (kaempferol diglycoside, rutin, kaempferol rhamnoside xyloside, diacetylafzelin, pentahydroxyflavone, hemslyanoside, galangin), anthocyanins (delphinidin (glucoside), cyanidin (coumaroyl glucoside), delphinidin coumaroyl glucoside, and cyanidin coumaroyl glucoside), and gingerenone A showed Pearson’s positive coefficient correlation value (0 < r < 1) with antioxidant activity and TFC assay. Anthocyanins are well known to be responsible for the color in plants and their relationship as antioxidants. Contents of anthocyanins in the aboveground part of *Z. officinale* gave the high antioxidant activity [34,44]. Gingerenone A as class of diarylheptanoid possessed antioxidant activity and was plentiful in the aboveground part of *Z. officinale*, contributing to its antioxidant activity [43].

Diarylheptanoid (7-(3,4-dihydroxy-5-methoxyphenyl)-5-hydroxy-1-(4-hydroxy-3-methoxyphenyl)-3-heptanone or 1-(3,4-dihydroxy-5-methoxyphenyl)-5-hydroxy-7-(4-hydroxy-3-methoxyphenyl)-3-heptanone), tetrahydrocurcumin, gingerenone A, and galanganol C showed Pearson’s positive coefficient correlation value (0 < r < 1) correlations with antioxidant activity and TPC assay (Figure 4). The highest antioxidant activity was observed in the root part of *Z. officinale* due to the presence of metabolites such as tetrahydrocurcumin and diarylheptanoid which have antioxidant activity showing highly positive correlation [45,46]. Precursor (phenylalanine) relative contents were high in the aboveground parts of *Zingiber* species. The metabolites majorly contributing to the highest antioxidant activity seen in the root parts of *Z. officinale* were tetrahydrocurcumin, galanganol C, and diarylheptanoid synthesized from the precursor but showing its highest relative contents in the root part. This result was due to the enzyme phenylalanine ammonia lyase (PAL) responsible for the formation of the gingerols and curcuminoids that exist in high levels in the plants of the Zingiberaceae family related with phenylpropanoid biosynthesis pathway [41]. Starting from this PAL enzyme, other enzymes like *p*-coumaroyl shimikate transferase and *p*-coumaroyl quinate transferase, which are involved with the synthesis of polyphenols and curcuminoids exist in high levels in the root part of *Zingiber* plants. High antioxidant activity correlated with the TPC assay and this can be attributed to enzymes in the root part of *Z. officinale*, which led to the production of secondary metabolites possessing antioxidant properties. Oils and other metabolites showed negative coefficient correlation values (−1 < r < 0) with antioxidant activity. It has already been reported that the polyphenols have antioxidant activity, but in our experiment 8-gingerol and 8-paradol showed negative coefficient correlation values with antioxidant activity, a result that didn’t match with the previous studies [42]. The small relative contents of 8-gingerol and 8-paradol which are long chain forms of gingerol and its derivative, respectively, in the root parts of *Z. mioga* may be responsible for this result. Each species and part showed different bioactivities and these could be attributed to the differences in relative contents of the identified metabolites. In particular, the root part containing polyphenols and aboveground part with flavonoids of *Z. officinale* showed the highest antioxidant activity. Based on these results, identifying the metabolites with differential bioactivities between *Zingiber* species and structural parts may lead to new and more efficient uses for the different species and parts. 

## 3. Experimental Section 

### 3.1. Chemicals and Reagents

Methanol, acetonitrile, hexane, and water were purchased from Fisher Scientific (Pittsburgh, PA, USA). Methoxyamine hydrochloride, *N*-methyl-*N*-(trimethylsilyl) trifluoroacetamide (MSTFA), diethylene glycol, gallic acid, naringin, 6-hydroxy-2,5,7,8-tetramethylchroman-2-carboxylic acid (Trolox), hydrochloric acid, potassium persulfate, 2,2″-azinobis (3-ethylbenzothiazoline-6-sulfonic acid) diammonium salt (ABTS), 1,1-diphenyl-2-picrylhydrazyl (DPPH), formic acid, pyridine, and standard compounds were obtained from Sigma Chemical Co. (St. Louis, MO, USA). All other chemicals used in the experiment were of analytical grade.

### 3.2. Plant Materials

All the samples of *Z. mioga* and *Z. officinale* were provided by the Korea Plant Extract Bank. The subterranean parts which are called the root or rhizome, are classified as being identical in both *Zingiber* species. (*Zingiber mioga* Thunb. Root part; ZMR and *Zingiber Officinale* Thunb. Root part; ZOR). The aerial part of *Z. mioga* and *Z. officinale* included the leaf, stem, and flower (*Zingiber mioga* Thunb. Aboveground part; ZMA and *Zingiber Officinale* Thunb. Aboveground part; ZOA). However, the aerial parts differ slightly between species. The flowers of *Z. mioga* are the young flower bud part before if blooms, whereas the flower of *Z. officinale* is the mature floral leaf after it has bloomed. The aerial and subterranean parts of *Z. mioga* were collected in March 2002 and June 2002. The aerial and subterranean parts of *Z. officinale* were both collected in November 2008. The samples were extracted with 99.9% methyl alcohol and stored at −80 °C after drying.

### 3.3. Procedures for Preparation of Sample Solution

For gas chromatography−time-of-flight-mass spectrometry (GC-TOF-MS) analysis, two derivatization steps were performed. First, oximation was conducted by dissolving the dried extracts in 50 μL of methoxyamine hydrochloride (20 mg/mL in pyridine) and reacting at 30 °C for 90 min. The samples were then silylated with 50 μL of MSTFA at 37 °C for 30 min. For ultraperformance liquid chromatography−quadrupole-time-of-flight-mass spectrometry (UPLC-Q-TOF-MS) analysis, dried extracts of the *Zingiber* samples (20 mg/mL) were resolved with methanol and filtered through a 0.2 μm PTFE filter. Three analytical replications were performed for the GC-TOF-MS and UPLC-Q-TOF-MS analyses.

### 3.4. Instrumental Analysis

#### 3.4.1. GC-TOF-MS Analysis

An Agilent 7890A GC system (Palo Alto, CA, USA) equipped with an Agilent 7693 autosampler was attached to a TOF Pegasus III mass spectrometer (Leco, St. Joseph, MI, USA), operating in electron ionization (EI) mode (70 eV). A DB-5MS column (29.8 m length × 0.25 mm i.d. × 0.25 m film thickness, J & W Scientific, Folsom, CA, USA) was used with helium as a carrier gas at a constant flow of 1.5 mL/min. Then, 1 μL of the derivatized sample was injected in a splitless mode. The oven temperature was maintained at 75 °C for 2 min, increased to 300 °C at a rate of 15 °C/min, and then held at 300 °C for 3 min. The voltage of the detector was 1450 V. The acquisition rate was set to 10 scans/s and a mass scan range was set from 45 to 1000 *m*/*z*. The injector and ion source temperatures were 250°C and 230°C, respectively.

#### 3.4.2. UPLC-Q-TOF-MS Analysis

A Waters ACQUITY UPLC system (Waters Corp., Milford, MA, USA) equipped with a binary solvent delivery system, an autosampler, and a UV detector was combined with a Waters Q-TOF Premier MS (Micromass MS Technologies, Manchester, UK) system. Aliquots (5 μL) of each sample were then injected into an ACQUITY BEH C18 column (100 mm × 2.1 mm i.d. × 1.7 μm particle size) at a flow rate of 0.3 mL/min. The elution was performed along an acetonitrile/water gradient containing 0.1% formic acid. The gradient was linearly increased from 5% to 100% acetonitrile over 10 min and then decreased to 5% over 2 min. The total run time, including re-equilibration of the column to the initial conditions, was 14 min. The mass spectrometer was operated in both negative and positive ion modes with an m/z range of 100−1000. The desolvation gas (nitrogen) was set to 700 L/h at a temperature of 300 °C. The cone gas (nitrogen) was set to 0.00 L/h, and the source temperature was 100 °C. The capillary and cone voltages were set to 2.5 kV and 30 V, respectively. Data were collected in the centroid mode, with a scan accumulation time of 0.2 s. All analyses were performed using an independent reference spray via the LockSpray interference to ensure accuracy and reproducibility; Leucine enkephalin ions were used as the lock mass (*m*/*z* 554.2615 [−] and 556.2771 [+]) at a flow rate of 10 μL/min. Accurate mass and elemental composition were calculated using the MassLynx software (Waters Corp.) incorporated in the instrument.

### 3.5. Data Processing and Statistical Analysis 

GC-TOF-MS raw data were converted to netCDF format with ChromaTOF software (LECO). UPLC-Q-TOF-MS raw data were acquired with MassLynx software and converted into netCDF format with Waters DataBridge version 2.1 software. After conversion, the MS data were processed using the Metalign software package (http://www.metalign.nl). The resulting data were exported to Excel (Microsoft, Redmond, WA, USA), and a statistical analysis was performed using SIMCA-P+ 12.0 software (Umetrics, Umea, Sweden). Principal component analysis (PCA) was used to compare the differences between *Z. officinale* and Z. *mioga* with their root and aboveground parts to identify the major significant metabolites. The data sets were unit variance scaled in a column-wise manner prior to PCA modeling. Metabolites with a variable importance projection (VIP) value greater than 0.7 and a *p*-value less than 0.05 were selected as potential metabolites that could be differentially produced in the two species. Differences in total phenolic content (TP), total flavonoid content (TF), FRAP, DPPH, and ABTS radical scavenging activities were tested by analysis of variance and Duncan’s multiple range test using SPSS version 12.0 software (SPSS Inc., Chicago, IL, USA). Relative contents of significantly different metabolites and antioxidant activities were represented by Pearson’s correlation coefficient.

### 3.6. Determination of Antioxidant Activities by ABTS, DPPH, and FRAP Free Radical Scavenging Activity 

For the ABTS assay, we followed the method of Re *et al.* with some modifications [47]. The stock solutions included 7 mM ABTS solution and 2.45 mM potassium persulfate solution. The working solution was prepared by mixing the two stock solutions in equal quantities and allowing them to react for 1 day at room temperature in the dark. The solution was then diluted until the absorbance reached 0.7 ± 0.02 at 734 nm by using a spectrophotometer (Spectronic Genesys 6, Thermo Electron, Madison, WI, USA). Each diluted sample of *Zingiber* extract (20 μL) was reacted with 180 μL of the diluted ABTS solution for 7 min in the dark. Absorbance was then measured at 734 nm using a spectrophotometer. The standard curve was linear between 0.0625 and 1 mM Trolox equivalents (TE). The results are expressed in millimolar TE per milligram of dry weight of extract (ext).

The DPPH assay was conducted according to the method described by Dietz *et al.* with some modifications [48]. Diluted samples of *Zingiber* extracts (20 μL) were reacted with 180 μM DPPH solution for 20 min at room temperature. Absorbance was then measured at 515 nm. The standard curve was linear between 0.015 and 2 mM TE. Experiments were conducted in triplicate. For the FRAP assay [49], a mixture of 10 mM TPTZ solution in 40 mM HCl, 20 mM iron (III) chloride, and 300 mM acetate buffer at pH 3.6 (1:1:10, *v*:*v*:*v*) was made as FRAP reagent. For the analysis, 300 μL of FRAP reagent and 10 μL of sample (2-fold dilution in 100% methanol), were placed in a 96-well microplate and incubated at room temperature for 6 min. The absorbance was measured at 570 nm, and the results are expressed in mmol of Trolox equivalents concentration/mg dry weight basis. Trolox (0.015–2 mM) served as standards to quantify the antioxidant activity of the samples.

### 3.7. Total Phenolic Content and Total Flavonoid Content

Total phenolic content (TPC) in *Zingiber* family samples was determined according to the Folin−Ciocalteu colorimetric method, as modified by Singleton *et al.* [50]. Briefly, 0.2 N Folin−Ciocalteu’s phenol reagent (100 μL) was added to 20 μL of each sample placed in 96-well plates, followed by incubation in the dark for 5 min. After that, 7.5% sodium carbonate solution (80 μL) was added to the mixture, which was then measured at 750 nm using a microplate reader (Thermo Electron Spectronic Genesys 6). TPC was calculated on the basis of a standard curve with gallic acid (GA) equivalent concentration (ppm). The standard solution concentration curve ranged from 15.625 to 2000 ppm. All experiments were conducted in triplicate. Total flavonoid content (TFC) was measured, and 180 μL of 90% diethylene glycol, 20 μL of 1 N NaOH, and 20 μL of each sample were then mixed and incubated for 60 min at room temperature in the dark. Absorbance was measured at 405 nm using a microplate reader (Thermo Electron Spectronic Genesys 6). The results are presented as naringin (NG) equivalent concentration (ppm). The standard solution concentration curve ranged from 15.625 to 2000 ppm. All experiments were conducted in triplicate. 

## 4. Conclusions

*Z. officinale* and *Z. mioga* with root and aboveground parts were shown to be discriminated by their different levels of metabolites and bioactivities determined using untargeted and unbiased metabolomics. The different relative contents of metabolites in species and parts resulted in different antioxidant activities. Those profiled metabolites simultaneously were biomarkers to discriminate usage depending on different species and parts. This approach is not limited only to *Zingiber* species and could be effectively applied to other edible food plants.

## Supplementary Materials

Supplementary materials can be accessed at: http://www.mdpi.com/1420-3049/20/09/16170/s1.
