# Supplementary material for: MS-Based Metabolite Profiling of Aboveground and Root Components of Zingiber mioga and Officinale
Source: Molecules. 2015 Sep 3;20(9):16170–85. doi: 10.3390/molecules200916170 (PMC6332244; doi:10.3390/molecules200916170)
Supplement: Supplementary file 1 [file molecules-20-16170-s001.pdf]

Supplementary Information

Table S1. Differential Metabolites Identified Using GC-TOF-MS in *Z. officinale* and *Z. mioga* with their different parts.

No.	TR(min) [a]	Unique Mass [b]	Tentative Metabolite [c]	Derivatized [d]	MS Fragmentation Pattern [e]	ID [f]
			Organic Acids			
1	4.99	173	Hexanoic acid *	x3	58,117,173	MS
2	5.04	134	Acetic acid *	x3	73,103,134,177,205	STD,MS
3	5.16	72	Pyruvic acid *	x6	72,107,134,147,184,200	STD,MS
4	5.87	191	Propanoic acid ***	x2	100,103,117,177,191,000	MS
5	5.92	145	Butanoic acid *	x3	45,100,145,187,219	MS
6	6.02	130	D-2-Aminobutyric acid *	x3	73,117,130,177,207	STD,MS
7	6.15	211	Phosphoric acid **	x3	103,133,163,211,241	STD,MS
8	6.76	179	Benzoic acid *	x1	77,119,179,189	MS
9	7.24	91	Phenylacetic acid *	x2	59,91,117,142,174,180	STD,MS
10	7.33	247	Succinic acid *	x3	45,73,129,172,247	STD,MS
11	7.62	99	Fumaric acid *	x3	99,115,171,184,218,200	STD,MS
12	8.14	129	Glutaric acid **	x3	73,97,129,203,233,261	MS
13	9.02	189	meso-Erythritol **	x3	86,100,174,201,243	STD,MS
14	9.36	189	Pentitol *	x3	117,189,217,307	MS
15	9.47	219	Glycerol **	x3	117,189,219,246,321	MS
16	10.14	117	Ethylmalonic acid ***	x3	55,95,117,201,257	STD,MS
17	11.45	133	Citric acid ***	x4	133,157,183,273,375	STD,MS
			Amino Acids			
18	7.31	277	Glycine *	x3	45,73,129,174,247,277	STD,MS
19	7.85	306	Serine ***	x3	100,188,204,278,306	STD,MS
20	9.19	232	L-Aspartic acid *	x3	100,147,202,232	STD,MS
21	9.39	292	L-Threonic acid *	x4	117,189,220,292	STD,MS
22	10.04	328	Phenylalanine **	x2	100,192,218,266,328	STD,MS
23	10.38	258	L-Asparagine **	x3	116,188,231,258	STD,MS
24	12.25	100	L-Tyrosine *	x3	103,179,218,280,319	STD,MS
			Fatty Acids			
25	13.85	337	Linoleic acid *	x1	95,129,178,220,262	STD,MS
26	13.91	108	α-Linolenic acid **	x1	73,93,108,129,191,335	STD,MS
27	14.01	341	Stearic acid *	x1	95,201,313,341	STD,MS
28	15.12	369	Arachidic acid *	x1	73,117,204,341,369	STD,MS
29	15.89	459	Palmitic acid *	x2	203,239,313,371,459	STD,MS
			Sugars			
30	11.88	307	D-Tagatose *	x6	103,189,217,277,307	STD,MS
31	12.07	320	D-Mannose **	$(MEOX)_{x2}$ x5	73,103,147,205,319	STD,MS
32	12.21	319	D-Galactose *	$(MEOX)_{x2}$ x5	73,103,147,205,319	STD,MS
33	12.97	277	L-Fucitol *	x3	117,191,217,277,319	STD,MS
34	14.58	204	Glyceryl-glycoside *	x6	103,204,243,305,337	MS

[a] TR(min) is the retention time. [b] Unique Mass are the selected ions(s) and quantification of individual derivatized metabolites. [c] Identified metablites based on the STD. [d] Derivatized MEOX, methlyoxime; TMS, trimethlysilyl. [e] MS fragmentation is the fragmentaion of Tentative compound. Identification: STD mass spectrum was consistent with those of standard compounds: Highest similarity with the Hit list. In the tentative compounds, * represents the metabolites differentiated by VIP value 1, ** represents metabolites differentiated both by VIP value 1 and 2, and *** represents metabolites differentiated by VIP value 2. All metabolites are selected by Vip > 0.7 & p-value < 0.05.

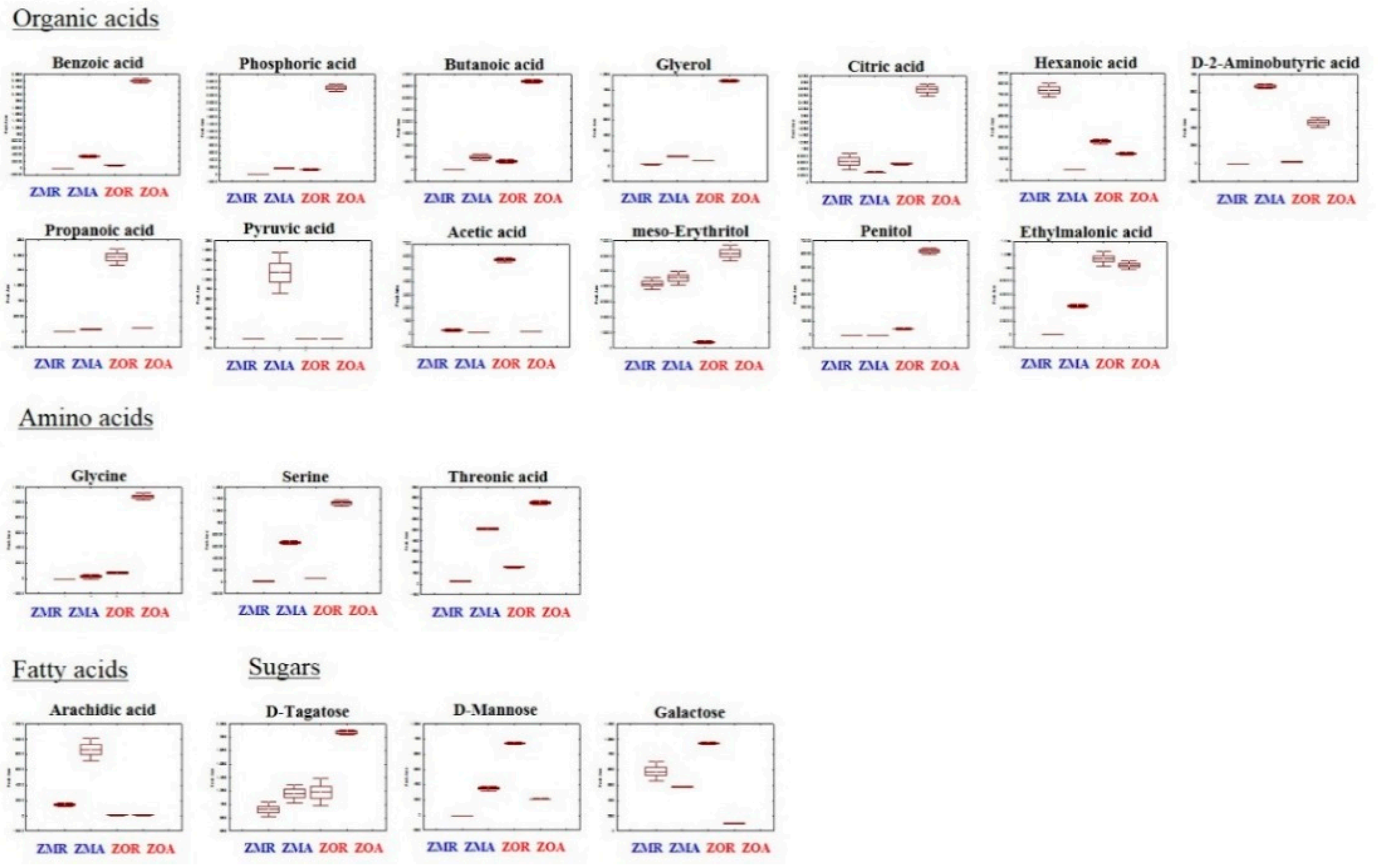

Figure S1. Box and whisker plots of significantly different metabolites between *Z. officinale* and *Z. mioga* with their different parts analyzed by GC-TOF-MS.

Table S2. Differential Metabolites Identified Using UPLC-Q-TOF-MS in *Z. officinale* and *Z. mioga* with their different parts.

No.	Vip	T_R (min)	Tentative Compound	Measured Mass (*m*/*z*)		Elemental Composition	*i*-FIT(norm)	Error(ppm)	Ref.
				$[M+H]^+$	$[M-H]^-$				
			Amino Acids						
1	1.24	1.29	Isoleucine ***	132.1014	130.0880	$C_6H_{13}NO_2$	0	6.9	CCD
2	1.39	2.04	Phenylalanine ***	166.0870	164.0720	$C_9H_{11}NO_2$	0	4.3	CCD
3	1.35	2.10	2-(Methylamino)benzoic acid ***	166.0898	164.0720	$C_9H_{11}NO_2$	0	3.7	CCD
4	1.08	2.88	Tryptophan ***	188.0724	203.0840	$C_{11}H_{12}N_2O_2$	0.1	−2	CCD
			Flavonoids						
5	1.28	3.15	Kaempferol diglycoside ***	623.1606	621.1471	$C_{28}H_{30}O_{16}$	2	0.2	[33]
6	1.35	3.64	Delphinidin (glucoside) ***	303.0526	N.D.	$C_{15}H_{10}O_7$	0.1	3	[34,36]
7	1.33	3.64	Rutin ***	611.1616	609.145	$C_{27}H_{30}O_{16}$	0.1	−1	CCD
8	1.30	3.66	Kaempferol rhamnoside xyloside ***	565.1570	563.1428	$C_{26}H_{28}O_{14}$	2.1	−1.2	[33]
9	1.30	3.79	Cyanidin (coumaroyl glucoside) ***	287.0574	N.D.	$C_{15}H_9O_6$	1.7	1	[33,34,37]
10	1.30	3.85	Delphinidin coumaroyl glucoside ***	611.1616	609.1450	$C_{27}H_{30}O_{16}$	0.1	−1	[33,34,37]
11	1.30	3.87	Diacetylafzelin ***	517.1384	N.D.	$C_{25}H_{24}O_{12}$	0	0	CCD
12	1.47	3.91	Pentahydroxyflavone ***	303.0532	301.0356	$C_{15}H_{10}O_7$	0.5	7.9	CCD
13	1.54	4.12	Cyanidin coumaroyl glucoside ***	595.1741	593.1510	$C_{30}H_{27}O_{13}$			[33,34,37]
14	1.24	4.17	Hemsleyanoside ***	549.1615	547.1489	$C_{26}H_{28}O_{13}$	0.2	−3.1	CCD
15	1.30	4.28	Galangin ***	271.0633	134.0372 (M-2H)	$C_{15}H_{10}O_5$	0.3	10	CCD
			Oils						
16	0.89	5.84	Trihydroxyoctadecenoic acid ***	N.D	329.1380	$C_{18}H_{34}O_5$	2.1	1.2	CCD
17	1.29	6.63	Aminohexadecanediol **	274.2764	N.D	$C_{16}H_{35}NO_2$	0	0.4	CCD
18	1.24	7.22	Aminooctadecenetriol *	316.2856	N.D	$C_{18}H_{37}NO_3$	0	−1.6	CCD
19	1.82	7.47	Aminooctadecanediol **	302.3070	N.D	$C_{18}H_{39}NO_2$	0	2.6	CCD
20	1.12	8.12	Oxo-octadecenoic acid ***	364.3234	N.D	$C_{23}H_{41}NO_2$	0	5.2	CCD
21	1.21	8.28	Aminoeicosanediol ***	330.3311	N.D	$C_{20}H_{43}NO_2$	0	0.6	CCD
22	1.47	8.41	Aminomethylnonadecanetriol ***	346.3339	N.D	$C_{20}H_{43}NO_3$	0	6.6	CCD
23	1.27	8.88	Aminoheneicosenediol ***	342.3327	N.D	$C_{21}H_{43}NO_2$	0.2	−2	CCD

No.	Vip	T_R (min)	Tentative Compound	Measured Mass (*m*/*z*)		Elemental Composition	*i*-FIT(norm)	Error(ppm)	Ref.
				$[M + H]^+$	$[M - H]^-$				
24	1.31	9.29	Halaminol A ***	228.2347	N.D	$C_{14}H_{29}NO$	0	3.1	CCD
25	1.26	9.50	Palmitoleamide ***	254.2499	N.D	$C_{16}H_{31}NO$	0	8.3	CCD
26	1.11	10.10	Palmitic acid *	N.D	255.2330	$C_{16}H_{32}O_2$	0	0.8	CCD
27	1.26	10.42	Oleamide *	282.2806	N.D.	$C_{18}H_{35}NO$	0	−2.5	CCD
			Others						
27	1.55	5.23	Diarylheptanoid **	N.D	389.1600	$C_{21}H_{26}O_7$	1.1	−5.9	CCD, [45]
28	1.23	5.81	[6]-Gingerdiol ***	353.2330	N.D	$C_{20}H_{32}O_5$	3.7	−2	CCD, [13]
29	1.40	5.84	Tetrahydrocurcumin [a]	373.1660	371.1461	$C_{21}H_{24}O_6$	0.1	3.2	CCD, [39,41]
30	0.90	5.85	Galanganol C ***	455.1699 (M + Na)	431.1740	$C_{23}H_{28}O_8$	0.4	−7	CCD
31	0.92	6.29	Gingerenone A ***	357.1679	355.1530	$C_{21}H_{24}O_5$	1	−1.7	CCD
32	0.92	7.16	Curcumadiol ***	N.D	237.1110	$C_{15}H_{26}O_2$	0.1	−3.4	CCD
33	0.82	7.62	[8]-Gingerol *	N.D	321.2050	$C_{19}H_{30}O_4$	2.8	−1.9	[12,13,46]
34	1.33	7.81	Labdene-diol *	441.3247	N.D	$C_{25}H_{44}O_6$	0.5	−2	CCD
35	1.47	8.61	Gingerglycolipid A **	677.3791	N.D	$C_{33}H_{56}O_{14}$	1.4	−3.5	CCD
36	1.80	8.72	[8]-Paradol *	N.D	305.2090	$C_{19}H_{30}O_3$	0	0.3	[13]

In the tentative compounds, * represents the metabolites differentiated by VIP value 1, ** represents metabolites differentiated both by VIP value 1 and 2, and *** represents metabolites differentiated by VIP value 2.Metabolites indentified by Dictionary of Natural Products are marked with CCD. N.D. represents for not detected. All metabolites are selected by Vip > 0.7 & *p*-value < 0.05.

Oil & Others

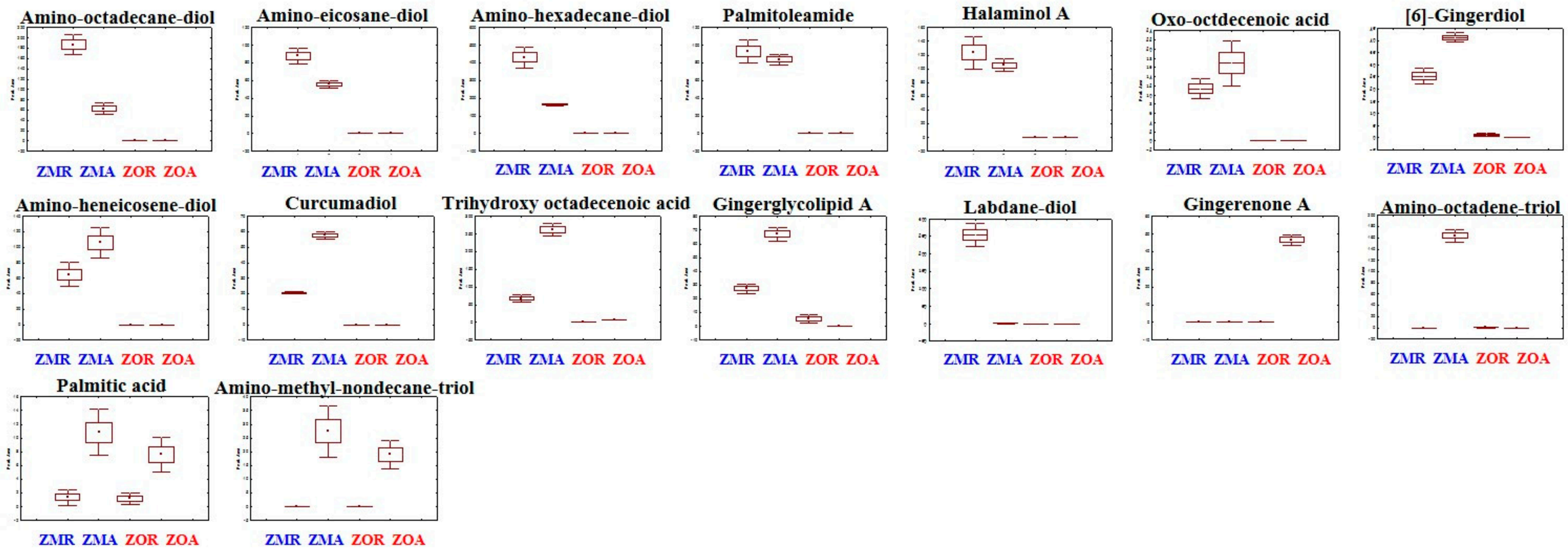

Figure S2. Box and whisker plot of significantly different metabolites between *Z. officinale* and *Z. mioga* with their different parts analyzed by UPLC-Q-TOF-MS.
